# Targeting fatty acid synthase to overcome PARP inhibitor resistance and to create an artificial synthetic lethality for triple-negative breast cancer

**DOI:** 10.1016/j.gendis.2025.101817

**Published:** 2025-08-20

**Authors:** Sophia Josephraj, Chao J. Wang, Qingbin Cui, Zizheng Dong, Jing-Yuan Liu, Jian-Ting Zhang

**Affiliations:** a Department of Cell and Cancer Biology, University of Toledo College of Medicine and Life Sciences, Toledo, OH 43614, USA; b Department of Medicine, University of Toledo College of Medicine and Life Sciences, Toledo, OH 43614, USA

**Keywords:** DNA damage repair, Fatty acid synthase, PARP inhibitor, Proton pump inhibitor, Synergy, TNBC

## Abstract

Despite advances in cancer treatment with targeted therapies and immunotherapies, triple-negative breast cancer (TNBC) has not significantly benefited from these developments. Although poly (ADP-ribose) polymerase (PARP) inhibitors (PARPi) are approved for breast cancer, their clinical use is largely limited to the small subset of HER2-negative patients with germline BRCA1/2 mutations, and resistance is frequently observed. Previously, we demonstrated that proton pump inhibitors (PPIs), including lansoprazole and its metabolite, 5-hydroxy lansoprazole sulfide (5HLS), reduce PARP1 expression by inhibiting fatty acid synthase (FASN), a key enzyme in *de*-*novo* lipid synthesis. We also found that PPIs synergize with DNA-damaging agents by regulating PARP1 expression and impairing non-homologous end joining (NHEJ) repair of DNA damage. These findings led to the hypothesis that PPIs synergize with PARPi independently of BRCA mutation, potentially expanding the utility of PARPi to a broader TNBC population. In this study, we show that FASN contributes to PARPi resistance, and that lansoprazole and 5HLS strongly synergize with olaparib and talazoparib in both BRCA1-mutant and wild-type TNBC cells. This synergy occurs through FASN inhibition and subsequent impairment of NHEJ repair of double-strand breaks induced by PARPi trapping. 5HLS also facilitates PARPi-induced PARP1 trapping and inhibits BRCA1 expression by inhibiting FASN, contributing to the synergy with PARPi in both BRCA1 wild-type and mutant TNBC cells. Together, these findings suggest that inhibiting FASN with PPIs creates an artificial synthetic lethality, providing a rationale for combining PPIs with PARPi to expand their utility to TNBC patients without germline BRCA1 mutations and to overcome PARPi resistance.

## Introduction

Breast cancer is the second most common malignancy and the leading cause of death among women worldwide.^1^ Triple-negative breast cancer (TNBC) accounts for 10%–20% of all breast cancer cases and is known for its aggressive nature, with significantly lower five-year survival rates and poorer prognoses than other subtypes of breast cancer.^2^, ^3^, ^4^ Furthermore, TNBC patients have not benefited much from recent therapeutic advancements, including targeted and immunotherapies. Standard-of-care chemotherapy, including adriamycin, cyclophosphamide, and taxanes, remains the first-line neoadjuvant treatment for TNBC.

While poly (ADP-ribose) polymerase (PARP) inhibitors (PARPi), such as olaparib and talazoparib, have been approved for human epidermal growth factor receptor 2 (HER2)-negative metastatic/advanced breast cancer patients with germline breast cancer susceptibility gene 1/2 (BRCA1/2) mutations, their therapeutic impact on BRCA wild-type TNBC remains modest due to lack of synthetic lethality and inherent resistance mechanisms.^5^, ^6^, ^7^, ^8^ This limited efficacy of olaparib and talazoparib as monotherapies highlights the need for novel combination strategies by creating an artificial synthetic lethality.

Human fatty acid synthase (FASN), the sole cytosolic enzyme responsible for *de*-*novo* lipid synthesis in mammalian cells, is essential for cancer cell survival but not for normal cells due to sufficient dietary lipids.^9^ FASN has also been shown to contribute to resistance to DNA-damaging drugs and radiation^10^^,^^11^ by up-regulating PARP1 expression and non-homologous end joining (NHEJ) repair of DNA damage via specific protein-1 (SP1) and p65.^12^^,^^13^ The fact that FASN regulates PARP1 expression suggests that FASN may contribute to the cellular response to PARPi.

FASN has been considered a promising target for anti-cancer drug discovery due to its up-regulated expression in cancer cells and absence in most normal non-lipogenic tissues.^14^ In an effort to repurpose US FDA-approved drugs targeting FASN, proton pump inhibitors (PPIs) were found to effectively inhibit FASN by targeting its thioesterase domain and inhibit cancer cell proliferation.^15^ PPIs also synergize with DNA-damaging treatments by inhibiting PARP1 expression and NHEJ activity via FASN.^13^ Furthermore, as a metabolite of lansoprazole (a PPI), 5-hydroxy lansoprazole sulfide (5HLS), exhibited greater potency in inhibiting FASN activity and modulating PARP1-mediated NHEJ repair of oxidative DNA damage.^16^

The fact that FASN up-regulates PARP1 expression and that PPIs inhibit FASN, leading to a reduction in PARP1 expression and NHEJ activity, prompted us to hypothesize that PPIs may synergize with PARPi and create an artificial synthetic lethality in BRCA wild-type TNBC cells. In this study, we tested this hypothesis using lansoprazole and 5HLS in combination with olaparib and talazoparib in TNBC cells. We showed that FASN overexpression contributed to PARPi resistance and that the combination of FASN inhibitors (lansoprazole and 5HLS) with PARPi synergistically inhibits DNA damage repair activity, leading to synergized DNA damage accumulation and cell death of TNBC cells, irrespective of their BRCA1 status. We also showed that 5HLS facilitated PARPi-induced PARP1 trapping and inhibited BRCA1 expression via inhibiting FASN, creating an artificial synthetic lethality for the opportunity to combine with PARPi and expand PARPi utility to TNBC patients without BRCA1 germline mutation and overcoming PARPi resistance.

## Materials and methods

### Materials

Antibody against FASN (#610963) was purchased from Millipore (Kankakee, Illinois, USA). Antibodies against cleaved PARP1 (#9451) and histone H3 (#9715) were purchased from Cell Signalling (Danvers, Maryland, USA). Antibodies against PARP1 (#66520) and phosphorylated histone H2AX (γH2AX) (#613402) were from Proteintech (Rosemont, Illinois, USA) and BioLegend (San Diego, California, USA), respectively. The antibodies against actin (#MABT219) and anti-mouse secondary (#A2554) and anti-rabbit secondary (#A0545) antibodies were from Sigma (St. Louis, Missouri, USA). 5HLS was synthesized in-house and validated for its purity to 95%. Olaparib (#S1060) and talazoparib (#7048) were procured from Selleckchem (Houston, Texas, USA). Dual luciferase reporter assay system (#E1910) and Caspase Glo 3/7 assay kit (#G8091) were purchased from Promega (Madison, Wisconsin, USA). Cell culture media, including Minimum Essential Medium (MEM; #10-010-CV) and Dulbecco's Modified Eagle Medium (DMEM; #10-013-CV), fetal bovine serum (#A316061), and apoptosis annexin V assay kit (#AD12) were from Corning (Corning, New York), GIBCO (Waltham, Maryland, USA), and Dojindo Molecular Technologies (Rockville, Maryland, USA), respectively. The Bradford protein quantification assay kit (#5000006) and polyvinylidene difluoride (PVDF) membranes were purchased from Bio-Rad Laboratories (Hercules, California, USA). All other chemicals were of molecular biology grade from Fisher Scientific (Chicago, Illinois, USA) or Sigma (St Louis, Missouri, USA).

### Cell lines

TNBC cell line MDA-MB-231 was maintained in DMEM, and cell line MDA-MB-436 was cultured in MEM, both supplemented with 10% fetal bovine serum and 1% penicillin-streptomycin (#MT30002CI, Corning, New York, USA). All cell lines were maintained at 37 °C in a 5% CO_2_ atmosphere. All cell lines were validated through DNA profiling using short tandem repeat (STR) analysis, as well as assessments of morphology, cell viability, and mycoplasma contamination.

### Generation of stable cell lines

MDA-MB-231 and MDA-MB-436 cells were seeded in six-well plates at a density of 2 × 10^5^ cells/well and cultured overnight. For overexpression, these cells were transfected with 2 μg of either pcDNA3.1-FASN,^13^ pCMV3-CGFP-Spark/PARP1 (#HG11040-ACG, Sino Biological, Houston, Texas, USA), or their respective empty vector control using Lipofectamine™ 3000 (Invitrogen). For stable knockdown, the cells were transfected with pSilencer2.1-U6/shFASN^13^ or shPARP1 (#sc-29437, Santa Cruz, California, USA) plasmids using Lipofectamine™ 3000 (Fisher Scientific), according to the manufacturer's instructions. Forty-eight hours post-transfection, cells overexpressing FASN and PARP1 were selected with 800 μg/mL G418 and 100 μg/mL hygromycin B, respectively. FASN and PARP1 knockdown cells were selected using G418 (800 μg/mL) and puromycin (2 μg/mL), respectively. After selection, antibiotic-resistant stable cells were isolated, pooled, and expanded for subsequent experiments.

### Pharmacological treatments and methylene blue survival assay

Pharmacological treatments and methylene blue survival assay were performed as previously described.^16^, ^17^, ^18^ Briefly, 2000–5000 cells per well, depending on the cell line, were seeded in 96-well plates and incubated overnight. Cells were then treated with various agents individually or in combination every 24 h for a total duration of 72 h. Following treatments, surviving cells were fixed with methanol for 30 min, stained with 1% (w/v) methylene blue (Sigma, #M4159) in 10 mM borate buffer (pH 8.5) for 30 min, and rinsed three times with double-distilled water. The stain was subsequently extracted using a 1:1 mixture of 100% ethanol and 0.1 M HCl, and absorbance was measured at 650 nm. Concentration-dependent survival curves were analyzed using GraphPad software to derive IC_50_ values.

### Colony formation assay

The colony formation assay was performed as previously described.^13^^,^^15^ Briefly, 200–500 cells were seeded in 6-well plates and incubated for 24 h before treatments with 5HLS, olaparib, and talazoparib as single agent or in combination. Cells were maintained for 7–10 days with media and drugs replenished every other day. At the end of treatments, colonies were fixed and stained using a solution containing 20% (v/v) methanol and 0.05% (w/v) crystal violet. Colonies containing 50 or more cells were counted.

### Subcellular fractionation and western blotting analysis

To isolate total cell lysate for western blotting analysis, cells were washed with phosphate-buffered saline and lysed in Tris-NaCl-NP40 (TNN) buffer as reported earlier,^16^ and protein concentrations were measured using Bradford reagents. To isolate nuclear and chromatin fractions, the subcellular protein fractionation kit (#78840, ThermoFisher Scientific) was used according to the manufacturer's instructions. Briefly, cells were washed with phosphate-buffered saline and incubated in the provided lysis buffer at 4 °C for 10 min with gentle mixing, followed by various steps of centrifugation to isolate nuclei, which were then used for isolation of nuclear soluble and chromatin-bound proteins as instructed.

Western blotting analysis was conducted as previously described.^19^ Briefly, an equal amount of protein was separated by SDS-PAGE and transferred onto PVDF membranes, which were blocked with 5% milk in Tris-buffered saline with Tween 20 (TBST) for 2 h, followed by incubation with primary antibodies diluted in 5% milk in TBST for 4 h or overnight. After three washes (15 min each) in TBST, membranes were incubated with a secondary antibody diluted in 5% milk in TBST for 1 h, followed by three 15-min washes in TBST. The signals were developed using enhanced chemiluminescence and captured using X-ray films.

### Apoptosis assay

The Caspase-Glo® 3/7 assay was performed following the manufacturer's protocol. Briefly, MDA-MB-231 and MDA-MB-436 cells were seeded in 96-well plates at a density of 5000–10,000 cells per well. After overnight incubation, cells were treated with DMSO vehicle control, 5HLS, talazoparib, or the combination for 24 h, followed by the addition of caspase-3/7 reagent according to the manufacturer's instructions and incubation at room temperature for 1 h. Luminescence was then measured using a SpectraMax iD5 microplate reader (Molecular Devices, San Jose, California, USA).

Annexin V staining assay was performed as previously described.^20^^,^^21^ Briefly, MDA-MB-231 and MDA-MB-436 cells were seeded in a 96-well black microplate with a clear bottom (2 × 10^4^ cells per well) and cultured overnight. The cells were then treated with DMSO, 5HLS, talazoparib, or the combination for 48 h, followed by incubation with annexin V-FITC and quenching buffer at room temperature for 15 min. Fluorescence intensity was measured using a BiTek Cytation 5 plate reader (Agilent Technologies, Winooski, Vermont, USA) with excitation at 488 nm and emission at 525 nm.

### NHEJ activity assay

The host cell reactivation assay of NHEJ activity was conducted as previously described,^12^^,^^13^ with minor modifications. Briefly, 5 × 10^4^ cells per well were seeded into 24-well plates and cultured overnight. After 48 h of treatments with DMSO, 5HLS, talazoparib, or the combination, cells were transfected with either 400 ng of intact (control) or Hind III-linearized (test) pGL3 plasmid encoding firefly luciferase (FL), along with 20 ng of pRL-TK plasmid (Promega) encoding renilla luciferase (RL) using Lipofectamine Plus. At 8 h post-transfection, cells were harvested, and both FL and RL activities were measured using the dual-luciferase reporter assay system. The FL activity in both control and test samples was normalized to RL activity, and NHEJ activity was calculated using the formula: NHEJ activity = normalized FL activity in the test group × 100/normalized FL activity in the control group.

### *In*-*vivo* efficacy

*In*-*vivo* studies using animals were approved by the IACUC at the University of Toledo. For *in vivo* efficacy assessment, 2 × 10^6^ MDA-MB-231 cells were injected into the mammary fat pad of 5-to-6-week-old female NSG mice. Once tumors reached approximately 50 mm^3^, the mice were randomized into four groups with five animals per group and treated with vehicle (group 1), 100 mg/kg 5HLS daily via intraperitoneal injection (group 2), 0.3 mg/kg talazoparib daily via oral gavage (group 3), and combination of 5HLS and talazoparib at the same dose and frequency (group 4) for 22 days. Mice were monitored daily, and body weight and tumor volume were measured three times a week. At the end of the treatments, mice were euthanized, and tumor tissues were collected for western blotting and immunohistochemical analyses.

### Immunohistochemistry analysis

Paraffin-embedded tissue sections were deparaffinized by heating at 80 °C for 10 min, followed by sequential washes in xylene, 100%, 95%, and 70% ethanol (5 min each), and rinsed in tap water. Antigen retrieval was performed by heating slides for 20 min in either citrate buffer (10 mM sodium citrate, 0.05% Tween-20, pH 6.0) or Tris–EDTA buffer (10 mM Tris, 1 mM EDTA, 0.05% Tween-20, pH 9.0). Slides were washed in TBST (0.05% Tween-20), blocked with 10% goat serum for 2 h, and incubated at 4 °C overnight with primary antibodies diluted in 10% serum. The slides were then rinsed in TBST, incubated with 0.3% hydrogen peroxide for 15 min, and incubated with secondary antibody for 1 h. After washing, slides were treated with Avidin–Biotin complex reagent for 30 min, followed by incubation with 3,3′-diaminobenzidine substrate (SigmaFAST™) for 1–3 min. Signal development was monitored microscopically, and excess DAB was neutralized with bleach. The tissue sections were then photographed using an Olympus VS120 microscope.

### TCGA dataset analysis

Analysis of the TCGA dataset was conducted as previously described using the R package.^22^ Briefly, the transcriptomic dataset for the Invasive Breast Carcinoma (TCGA-BRCA) cohort comprising 1103 primary tumor samples was obtained from cBioPortal (https://www.cbioportal.org/). Of these samples, 1089 had available expression data for both FASN and BRCA1. Gene expression values were derived from the Genomic Data Commons (GDC) and reported in transcripts per million (TPM). To normalize expression levels and reduce skewness, TPM values were transformed using log_2_(TPM + 1). To assess the relationship between FASN and BRCA1 expression, we calculated both Pearson correlation coefficients to evaluate linear relationships and Spearman rank correlations to assess monotonic trends. In addition, linear regression models were fitted to the log-transformed data to quantify the direction and magnitude of association in the TCGA-BRCA cohort.

### Synergy analysis

Cell-based synergy analyses were performed using either Isobole or the Chou-Talalay method for combination index (CI).^23^^,^^24^ If the combination IC_50_ data point is below the isobole of the additivity line in an isobologram or the CI is less than 1, the combination is synergistic. It is additive if the data point is on the isobole of additivity line or CI = 1, and antagonistic if the data point is above the isobole line or CI > 1.

For data with a single dose study, including the *in vivo* efficacy study, the synergy was analyzed using the Bliss independence model.^25^ Briefly, the expected combination additive inhibition (*I*_*C(E)*_) in % was calculated using the Bliss independence formula *I*_*C(E)*_ = *I*_*PPI(O)*_ + *I*_*PARPi(O)*_ – *I*_*PPI(O)*_ × *I*_*PARPi(O)*_, where *I*_*PPI(O)*_ and *I*_*PARPi(O)*_ represent observed (O) inhibition in percentage by PPI (lansoprazole or its derivative 5HLS) and PARPi (olaparib or talazoparib) as single agents, respectively. The combination is synergistic when the observed combination inhibition (*I*_*C(O)*_) is more than the calculated *I*_*C(E)*_, and additive if *I*_*C(O)*_ = *I*_*C(E)*_, and antagonistic if *I*_*C(O)*_ < *I*_*C(E)*_.

For cases where induction, rather than inhibition, was observed, including caspase activation and γH2AX expression, adapted Bliss independence model was used. For this purpose, fold-change (FC) values were first normalized to a fractional induction scale. Because the Bliss independence model operates on fractional effects ranging from 0 to 1, raw FC values were linearly transformed such that the untreated control with FC = 1 corresponds to zero induction, and the strongest observed induction (FC_max_) across all treatment groups corresponds to 1. The fractional induction or activation, f, was thus computed using the formula: f = (FC – 1)/(FC_max_ – 1) (here named the Bliss-Compatible Scaling Formula). This transformation preserves the relative spacing of induction levels across experimental conditions while constraining them to the unit interval [0, 1], thereby enabling valid application of the Bliss independence model. The resulting f values from single-agent treatment groups were then used to calculate the expected combination effect using the Bliss formula as described above.

### Statistical analysis

All statistical analyses were conducted using Prism GraphPad. A two-tailed *t*-test was used for comparing two groups, while ANOVA was applied for multiple group comparisons. Data were presented as mean ± standard deviation, typically from three independent experiments. A *P*-value of less than 0.05 was considered statistically significant. Statistical analyses were based on a minimum of three independent experiments and conducted using Excel or GraphPad Prism software.

## Results

### FASN contributes to PARPi resistance

**Figure 1:**
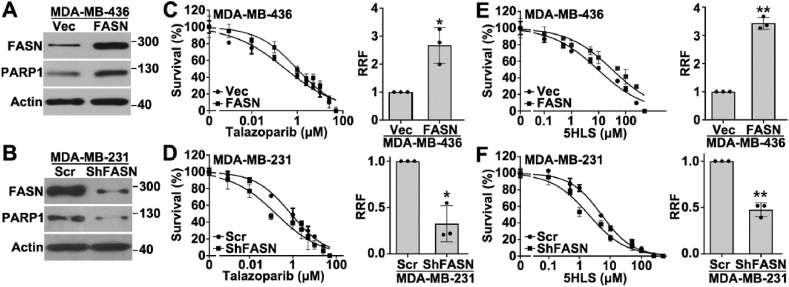
FASN's contribution to PARPi resistance. **(A, B)** Western blotting analysis of FASN and PARP1 expression in stable MDA-MB-436 cells overexpressing FASN (MDA-MB-436/FASN) and MDA-MB-231 cells with FASN knockdown (MDA-MB-231/shFASN) along with their respective control cells transfected with vector (Vec) and scrambled ShRNA (ShFASN). **(C–F)** Concentration-dependent survival curves of MDA-MB-436/FASN cells, MDA-MB-231/shFASN cells, and their respective control cells. Their relative resistance factor (RRF) against talazoparib and 5HLS was assessed using the methylene blue survival assay to derive IC_50_ values and calculated using the formula RRF = IC_50__(FASN or shFASN)_/IC_50(Vec or Scr)_. The data are presented as mean ± standard deviation. *n* = 3; ^∗∗^*P* < 0.01 and ^∗^*P* < 0.05.

To test the possibility that FASN expression may regulate cellular response to PARPi, we generated TNBC cell lines with stable FASN overexpression (Fig. 1A) or knockdown (Fig. 1B). These cells were then subjected to a survival assay in the presence of different concentrations of talazoparib. As shown in Figure 1C and D, MDA-MB-436 cells with FASN overexpression were significantly more resistant than the vector-transfected control cells, and MDA-MB-231 cells with FASN knockdown were significantly less resistant than the control cells harboring scrambled control shRNAs. The distinctive response of these cells with FASN overexpression or knockdown to 5HLS as a FASN inhibitor (Fig. 1E, F) confirmed that FASN function was altered by their expression level. Thus, we conclude that FASN expression and activity contribute to the cellular response to talazoparib.

### 5HLS and PARP inhibitors synergistically inhibit the growth of TNBC cells

**Figure 2:**
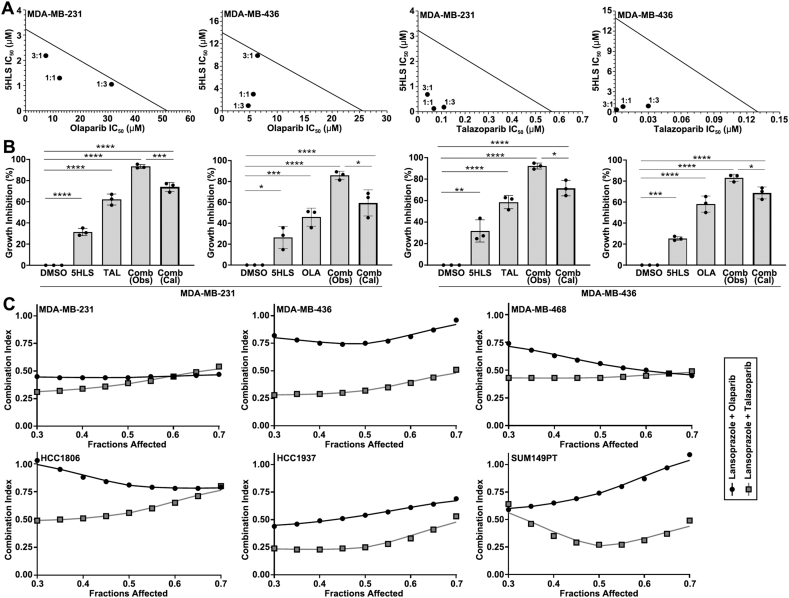
Synergism of PPI and PARPi combination in suppressing TNBC cell survival. **(A)** Isobologram analysis of the combination of 5HLS with olaparib or talazoparib in MDA-MB-231 and MDA-MB-436 cells using IC_50_ values of single agents and combinations derived from the methylene blue survival assay (Fig. S1, S2). The solid line represents the isobole of additivity connecting individual IC_50_ values of 5HLS, talazoparib, and olaparib as single agents derived from the survival assay. Each dot represents the result of a combination with 1:1, 1:3, or 3:1 5HLS/PARPi ratio, showing synergism under the isobole of the additivity line. **(B)** Bliss independence model analysis of combination synergy. Growth inhibition was calculated from the colony formation assay. **(C)** Synergism of lansoprazole's combination with olaparib or talazoparib with a 1:1 ratio, analyzed using single-agent and combination IC_50_ values from the methylene blue survival assay and the Chou-Talalay method. All data were from three independent experiments. ^∗^*P* < 0.05, ^∗∗^*P* < 0.01, ^∗∗∗^*P* < 0.001, and ^∗∗∗∗^*P* < 0.0001.

To validate the above findings and to evaluate the therapeutic potential of inhibiting FASN to overcome PARPi resistance, we performed combination analyses of 5HLS with olaparib or talazoparib. For this purpose, we first assessed the efficacy of each agent alone in inhibiting TNBC cell proliferation by performing a methylene blue survival assay of the parental MDA-MB-231 and MDA-MB-436 cells. As shown in Figure S1, 5HLS, olaparib, and talazoparib as single agents all dose-dependently inhibited the survival of MDA-MB-231 and MDA-MB-436 cells with IC_50_ values of 3.24 ± 0.43 to 13.97 ± 2.04 μM for 5HLS and 0.13 ± 0.05 μM to 51.57 ± 1.64 μM for PARPi, respectively. Next, we performed combination methylene blue survival analyses using 5HLS and PARPi with three different combination ratios (1:1, 1:3, and 3:1) based on their individual IC_50_ values (Fig. S1), followed by determining the combination IC_50_ value of each agent (Fig. S2). The isobologram analysis of the combination IC_50_ values showed that all combinations in different ratios resulted in strong synergism (Fig. 2A).

We also performed a colony formation assay of the combinations using one concentration for each inhibitor (Fig. S3), followed by analysis of the synergy using the Bliss independence model.^25^ As shown in Figure 2B, all combinations resulted in significant synergism, with the observed inhibition significantly greater than the expected combination additive effects according to the Bliss independence model. Based on the above results, we conclude that inhibiting FASN using 5HLS increases PARPi sensitivity.

### Lansoprazole synergizes with PARPi

To validate that the above synergism is from inhibition of FASN function, we tested lansoprazole, the parent compound of 5HLS, in combination with olaparib and talazoparib since lansoprazole has been shown to effectively inhibit FASN previously.^13^^,^^15^ We also tested additional TNBC cell lines for scientific rigor. Figure S4 shows the concentration-dependent survival inhibition of MDA-MB-231, MDA-MB-436, MDA-MB-468, HCC1806, HCC1937, and SUM149PT TNBC cells by lansoprazole (Fig. S4A), olaparib (Fig. S4B), and talazoparib (Fig. S4C). The derived individual IC_50_ values of each agent in all six cell lines are shown in Figure S4D. While the IC_50_ of lansoprazole was similar across all cell lines, that of olaparib and talazoparib varied dramatically among these cells. Interestingly, the IC_50_ values of olaparib and talazoparib did not appear to associate with the status of BRCA1 (Fig. S4E), which is consistent with prior observations.^26^

Lansoprazole was then combined with olaparib or talazoparib in a 1:1 ratio based on their individual IC_50_ values and used to treat the above six TNBC cell lines, followed by a methylene blue survival assay (Fig. S5). The combination IC_50_ values were then derived from these survival curves and used to analyze the combination index using the Chou-Talalay method.^23^ As shown in Figure 2C, all combinations with essentially all affected fractions resulted in synergistic inhibition in all six TNBC cell lines. Thus, we conclude that both lansoprazole and its metabolite 5HLS sensitize TNBC cells to PARPi and synergize with PARPi likely by inhibiting FASN.

### Effect of FASN and PARP1 expression on the combination synergism

**Figure 3:**
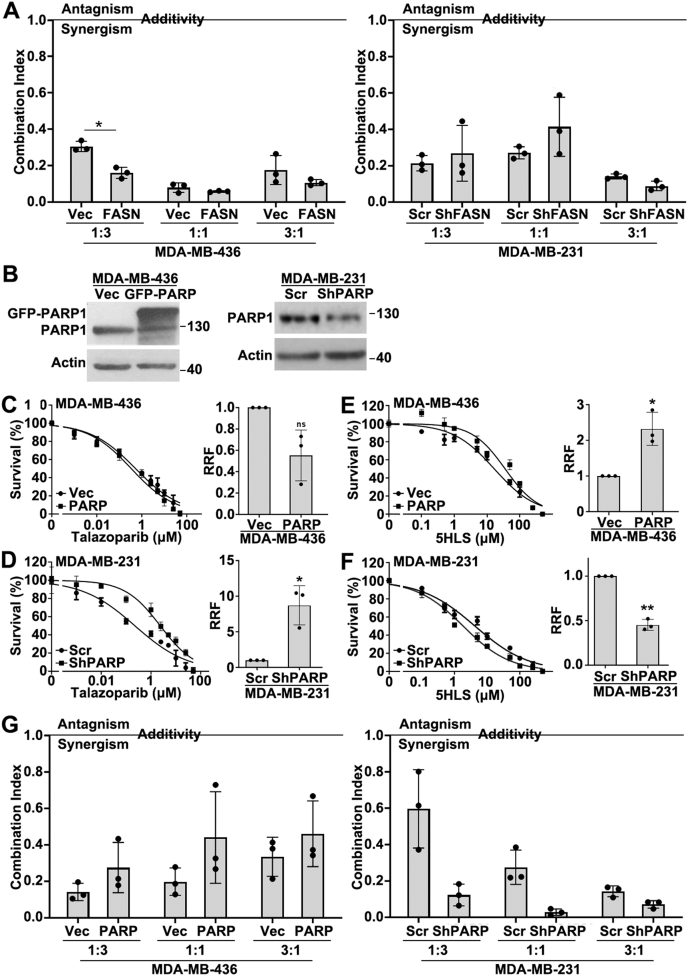
Effects of FASN and PARP1 expression on the combination synergy. **(A)** Combination index calculated using the Chou-Talalay method from combination survival analyses of FASN-overexpressing and FASN-knockdown cells using three different ratios of 5HLS and talazoparib. **(B–F)** Western blotting analysis of PARP1 and actin loading control (B) and analysis of methylene blue survival assay in the presence of talazoparib (C, D) or 5HLS (E, F) in GFP-PARP1-overexpressing MDA-MB-436 cells, PARP1-knockdown MDA-MB-231 cells, and their respective control cells. The relative resistance factor (RRF) was calculated using the formula: RRF = IC_50 (__PARP1 or sh__PARP1__)_/IC_50 (vec or scr)_. The data are presented as mean ± standard deviation. *n* = 3; ^∗^*P* < 0.05 and ^∗∗^*P* < 0.01. **(G)** Combination index calculated using the Chou-Talalay method from combination survival analyses of FASN-overexpressing and FASN-knockdown cells using three different ratios of 5HLS and talazoparib.

We next determined whether the expression level of FASN and PARP1 influenced the combination synergy observed above. For this purpose, we first took advantage of stable FASN overexpression and knockdown cells as described above (Fig. 1A) and performed the survival analysis (Fig. S6) using the combination between 5HLS and talazoparib with a 1:1, 1:3, or 3:1 ratio based on their individual IC_50_ values (Fig. 1). The combination IC_50_ values were then used to derive the combination index using the Chou-Talalay method. As shown in Figure 3A, the combination of 5HLS with talazoparib resulted in strong synergism with a combination index below 1. Depending on the ratio, FASN overexpression may reduce the combination index while FASN knockdown may increase it. However, this relationship mostly has no statistical significance.

Next, we established stable pooled MDA-MB-436 cells overexpressing GFP-PARP1 and MDA-MB-231 cells with PARP1 knockdown (Fig. 3B) and first determined their response to 5HLS and talazoparib individually as a single agent. As shown in Figure 3C and D, GFP-PARP1 overexpression reduced while its knockdown increased talazoparib resistance, consistent with the past findings and the role of PARPi in PARP1 trapping causing cytotoxicity.^27^ Interestingly, PARP1 overexpression increased and its knockdown reduced 5HLS resistance (Fig. 3E, F), consistent with our previous observations that the cytotoxicity of 5HLS is a result of its inhibition of FASN, leading to reduced PARP1 expression and increased oxidative DNA damage.^16^

Finally, the survival analyses of these stable cells were performed in the presence of 5HLS and talazoparib in combination with a 1:1, 1:3, or 3:1 ratio based on their individual IC_50_ values. As shown in Figure S7 and Figure 3G, these combinations all resulted in strong synergism with a combination index less than 1. In contrast to the findings with FASN overexpression and knockdown shown above, PARP1 overexpression appears to increase the combination index, while its knockdown could reduce it. Again, these trends have no statistical significance. Together with the above findings, we conclude that while the expression level of FASN and PARP1 significantly affects the cellular response to each agent individually, they also appear to affect the synergism between FASN inhibitors and PARPi, but to a lesser extent without statistical significance.

### The combination synergism on the induction of DNA damage and inhibition of NHEJ repair

**Figure 4:**
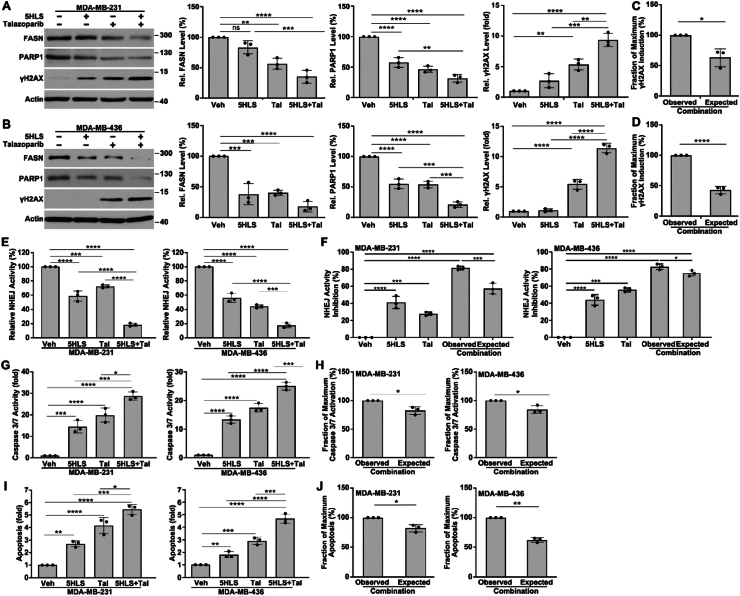
Combination synergy on DNA damage, NHEJ repair, and apoptosis. **(A, B)** Western blotting and quantification analyses of FASN, PARP1, γ-H2AX, and actin loading control in MDA-MB-231 and MDA-MB-436 cells treated with 5HLS, talazoparib, or the combination. **(C, D)** The fraction of maximum γ-H2AX induction derived using the Bliss-compatible scaling formula. **(E)** Host cell reactivation assay of NHEJ activity in MDA-MB-231 and MDA-MB-436 cells treated with 5HLS, talazoparib, or the combination. **(F)** Comparison between the observed and expected NHEJ activity inhibition by the combination using the Bliss independence model. **(G)** Caspasae3/7 activity assay of MDA-MB-231 and MDA-MB-436 cells treated with 5HLS, talazoparib, or the combination. **(H)** The fraction of maximum caspase 3/7 activation derived using the Bliss-compatible scaling formula. **(I)** Annexin V staining as an indicator of apoptosis in MDA-MB-231 and MDA-MB-436 cells following treatments with 5HLS, talazoparib, or the combination. **(J)** The fraction of maximum apoptosis induction derived using the Bliss-compatible scaling formula. *n* = 3; ^∗^*P* < 0.05, ^∗∗^*P* < 0.01, ^∗∗∗^*P* < 0.001, and ^∗∗∗∗^*P* < 0.0001.

To validate the above findings on the synergism, we evaluated the DNA damage induced by the combination. Because of the important role of PARP1 in DNA damage repair and its regulation by FASN, we hypothesized that the combination of 5HLS and talazoparib would have a synergistic effect on oxidative DNA damage by inhibiting the repair of the damage. To test this hypothesis, we treated the parental MDA-MB-231 and MDA-MB-436 cells with 5HLS and talazoparib as a single agent or in combination, followed by western blotting analysis of the expression level of γH2AX, a marker of double-strand DNA break.^28^ As shown in Figure 4A and B, 5HLS and talazoparib each individually induced the expression of γH2AX in both MDA-MB-231 and MDA-MB-436 cells, consistent with 5HLS's inhibition of FASN function in regulating DNA damage repair via PARP1^16^ (Fig. 4A, B), and talazoparib's inhibition of PARP1 catalytic activity and induction of PARP1 trapping.^29^^,^^30^ Interestingly, the combination of 5HLS and talazoparib induced significantly more production of γH2AX (Fig. 4A, B). Analysis of these data using the adapted Bliss independence model (see *Materials and Methods*) showed that the observed induction of γH2AX expression by the combination was strongly synergistic (Fig. 4C, D).

We next analyzed the combination effect on NHEJ repair activity in parental MDA-MB-231 and MDA-MB-436 cells. As shown in Figure 4E, both 5HLS and talazoparib significantly inhibited NHEJ activity, consistent with their function in inhibiting PARP1 expression and catalytic activity, respectively. The combination further reduced the NHEJ activity. Analysis of the combination data using the Bliss independent model indicated that the combination effect was synergistic in inhibiting NHEJ activity. This observation is consistent with the above finding that the combination synergistically induced γH2AX production. Thus, the combination of 5HLS and talazoparib synergistically inhibits NHEJ repair activity, leading to increased accumulation of DNA damage.

### The combination synergism on the induction of apoptosis

To determine if the synergistic effect on DNA damage repair leads to increased inhibition of cell survival, we next examined the combination effect on apoptosis induction in MDA-MB-231 and MDA-MB-436 cells by analyzing caspase 3/7 activity. As shown in Figure 4G, both 5HLS and talazoparib as a single agent induced significant activation of caspase 3/7. The combination, as expected, further induced caspase 3/7 activation. Analysis of the combination data using the adapted Bliss independence model showed that the combination synergized in inducing caspase 3/7 activation in both MDA-MB-231 and MDA-MB-436 cells (Fig. 4H).

To support the above findings, we performed apoptosis analysis using annexin V staining following treatments of MDA-MB-231 and MDA-MB-436 cells with 5HLS, talazoparib, or the combination. As shown in Figure 4I, both 5HLS and talazoparib induced significant apoptosis, and the combination induced significantly more apoptosis than any single agent alone. Analysis of the combination effect using the adapted Bliss independence model showed that the combination synergized in inducing apoptosis (Fig. 4J) in both cell lines. Together with the above findings, we conclude that the 5HLS-talazoparib combination synergistically inhibits DNA damage repair activities, leading to increased accumulation of DNA damage and induction of apoptosis.

### Synergy on PARP1 trapping

**Figure 5:**
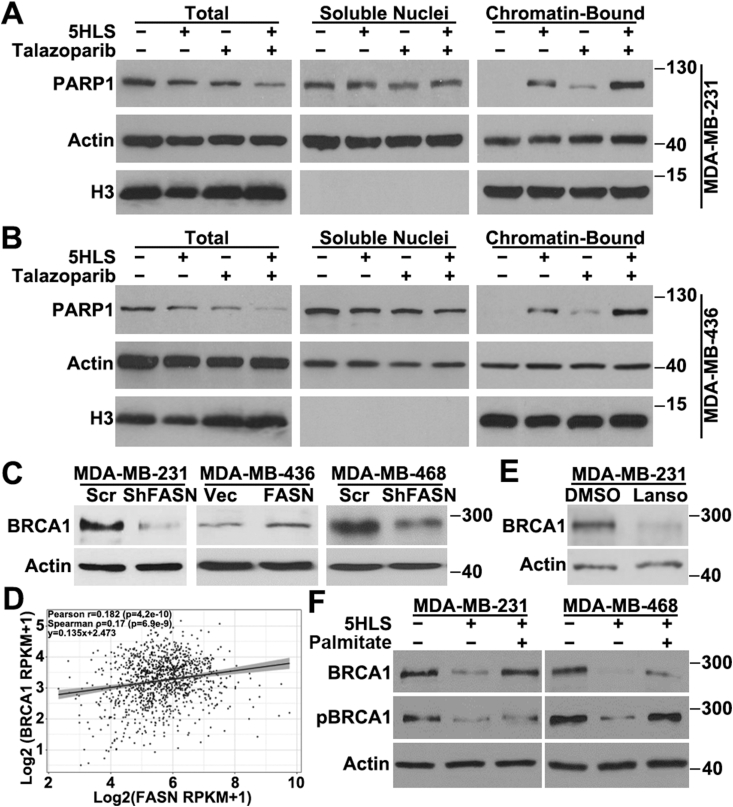
Effect of 5HLS on PARP1 recruitment and BRCA1 expression. **(A, B)** Western blotting and quantification analyses of PARP1 recruitment to chromatin and trapping, using isolated soluble nuclear and chromatin-bound fractions from MDA-MB-231 (A) and MDA-MB-436 (B) cells treated with vehicle, 5HLS, talazoparib, or the combination. **(C)** Western blotting analysis of BRCA1 and actin loading control in MDA-MB-436 cells with stable FASN overexpression, MDA-MB-231 and MDA-MB-468 cells with stable FASN knockdown, and their respective control cells. **(D)** Association between FASN and BRCA1 expression in the TCGA breast cancer dataset analyzed using the scatter plot of log_2_(FASN RPKM + 1) versus log_2_(BRCA1 RPKM +1). **(E)** Western blotting analysis of BRCA1 and actin loading control in MDA-MB-231 cells treated with lansoprazole and DMSO vehicle control. . **(F)** Western blotting analysis of BRCA1, pBRCA1, and actin control in MDA-MB-231 and MDA-MB-468 cells treated with vehicle or 5HLS in the absence or presence of excessive palmitate.

To understand the mechanism of 5HLS synergism with talazoparib, we analyzed the effect of 5HLS and talazoparib on PARP1 trapping in both MDA-MB-231 and MDA-MB-436 cells. For this purpose, chromatin-bound proteins from these cells following treatments with 5HLS, talazoparib, or the combination were subjected to western blotting analysis of PARP1. As shown in Figure 5A and B, talazoparib induced PARP1 recruitment to chromatin or trapping as expected. To our surprise, 5HLS alone also induced PARP1 recruitment to chromatin, although it reduced the total level of PAPR1 expression. The combination of 5HLS and talazoparib induced much more PARP1 recruitment than any single agent alone in both cell lines (Fig. 5A, B). These findings suggest that 5HLS may facilitate PARP1 recruitment to chromatin or its retention, enhancing talazoparib-induced PARP1 trapping and synergizing with talazoparib.

### FASN regulation of BRCA1 expression

The above findings on synergistic effects between PARPi and FASN inhibition in BRCA1 wild-type cells suggest that this combination may create an artificial synthetic lethality without a germline BRCA1 mutation. To investigate the potential mechanism leading to this artificial synthetic lethality, we tested the possibility that FASN may regulate BRCA1 expression. For this purpose, we first took advantage of the FASN-knockdown MDA-MB-231 cells and FASN-overexpressing MDA-MB-436 cells (see above) and tested BRCA1 expression using western blotting analysis. Figure 5C showed that BRCA1 expression was reduced in FASN-knockdown cells compared with the control cells, and, consistently, it was up-regulated in FASN-overexpressing MDA-MB-436 cells despite the fact that MDA-MB-436 cells express mutant BRCA1. Additionally, we took advantage of the stable MDA-MB-468 cells with FASN knockdown,^19^ which is known to express wild-type BRCA1, and showed that BRCA1 expression was reduced due to FASN knockdown (Fig. 5C).

To validate the above findings and to illustrate the clinical relevance of these findings, we performed co-expression analysis using the TCGA breast cancer patient cohort. As shown in Figure 5D, there was a significant positive association between FASN and BRCA1 expression with a Pearson correlation coefficient *r* = 0.26 (*P* = 2.49 × 10^−18^), a Spearman correlation coefficient ρ = 0.239 (*P* = 1.15 × 10^−15^), and the fitted linear regression y = 0.169x + 1.629. While the strength of the correlation was modest, typical for transcriptional data in large and heterogeneous cancer cohorts, the statistical robustness of this association adds independent evidence to support the conclusion that FASN may positively regulate BRCA1 expression, contributing to the above-observed synergism in BRCA1 wild-type or proficient TNBC cells.

To further validate the above findings and to determine if FASN activity is involved in regulating BRCA1 expression, we first treated the BRCA1 wild-type MDA-MB-231 cells with lansoprazole to inhibit FASN activity, followed by western blotting analysis of BRCA1. As shown in Figure 5E, BRCA1 was effectively reduced by lansoprazole treatment. Next, we treated both the BRCA1 wild-type MDA-MB-468 and MDA-MB-231 cells with 5HLS as another FASN inhibitor, followed by western blotting analysis of BRCA1 expression. As shown in Figure 5F, 5HLS inhibited the BRCA1 expression in both cell lines. Interestingly, supplementation of excess palmitate (the product of FASN) rescued BRCA1 expression from 5HLS inhibition, suggesting that 5HLS inhibition of BRCA1 expression is likely due to inhibition of FASN function.

Furthermore, we found that the basal level of Ser^988^-phosphorylated BRCA1 (pBRCA1 or activated BRCA1) was also inhibited by 5HLS, which was rescued by supplementation of excess palmitate (Fig. 5F). Taken together, these results suggest that FASN likely regulates BRCA1 expression and activation and that inhibiting FASN expression or function may create an artificial synthetic lethality with PARPi in BRCA1 wild-type TNBC cells.

### The *in*-*vivo* synergism between 5HLS and talazoparib

**Figure 6:**
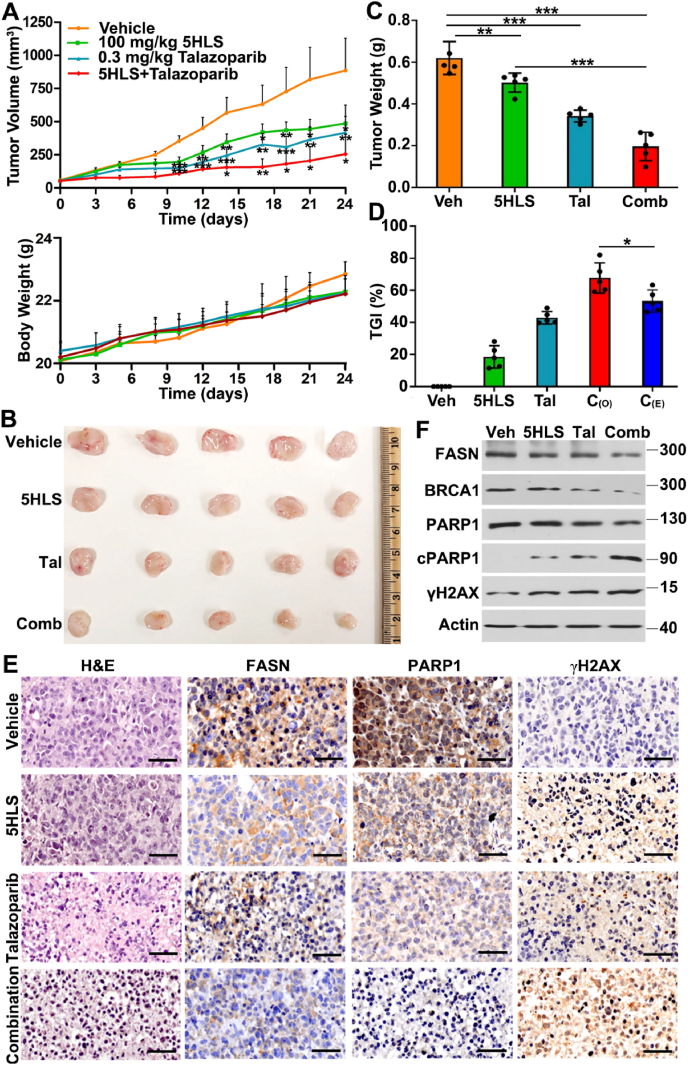
*In*-*vivo* effect of 5HLS and talazoparib combination on tumor growth. **(A)** Tumor volume and body weight of mice treated by vehicle (Veh), 5HLS, talazoparib (Tal), or combination (Comb) of 5HLS and talazoparib. **(B, C)** Gross anatomy (B) and wet weight (C) of dissected xenograft tumors at the end of the study. **(D)** Synergy analysis. Tumor growth inhibition (TGI) was derived using the wet weight of tumors. The expected combination inhibition (C_(E)_) was calculated from that of 5HLS and talazoparib alone using the Bliss independence model (see *Materials and Methods*). C_(O)_ represents the observed combination inhibition. **(E)** Immunohistochemical analyses of FASN, PARP, and γH2AX. Scale bar, 50 μm. **(F)** Western blotting analyses of FASN, BRCA1, PARP1, cleaved PARP1 (cPARP1), γH2AX, and actin loading control in xenograft tumors from mice treated with vehicle, 5HLS, talazoparib, and the combination of 5HLS and talazoparib. Each lane represents mixed samples of five tumors in equal proportion within the treatment group. *n* = 5; ^∗^*P* < 0.05, ^∗∗^*P* < 0.01, and ^∗∗∗^*P* < 0.001.

To assess the potential artificial synthetic lethality and overcome PARPi resistance by inhibiting FASN activity *in*-*vivo*, we tested the combination synergism between 5HLS and talazoparib on the BRCA1 wild-type TNBC xenograft tumor. Briefly, NSG mice bearing MDA-MB-231 xenograft tumors were randomized into four groups and treated for 22 days with vehicle control, 5HLS (100 mg/kg, intraperitoneal injection, once daily) as described previously,^16^ talazoparib (0.3 mg/kg, oral gavage, once daily) also as described previously,^31^ or both agents in combination. Tumor growth and body weight of the mice were monitored thrice a week. As shown in Figure 6A, the combination did not cause significant body weight loss compared with the control or single agent-treated groups, suggesting that there is no added toxicity from the combination. Furthermore, both 5HLS and talazoparib as single agents significantly inhibited the growth of xenograft tumors as expected. Also, as expected, the combination of 5HLS and talazoparib inhibited the tumor growth further. At the end of the study, xenograft tumors were dissected and weighed. Fig. 6B shows the gross anatomy of the dissected tumors and Figure 6C shows that the wet weight of xenograft tumors from all treatment groups is significantly less than the control group. Compared with the tumors in the single-agent-treated groups, the tumors in the combination group were significantly less in wet weight. Analysis of the tumor weight data using the Bliss independence model revealed that the combination of 5HLS and talazoparib was synergistic in inhibiting tumor growth (Fig. 6D).

To determine the *in*-*vivo* target inhibition, we performed immunohistochemistry staining of FASN, PARP1, and γH2AX in the xenograft tumors of all four groups. As shown in Figure 6E, 5HLS reduced the expression of FASN and PARP1, but increased the expression of γH2AX. This observation is consistent with our previous findings^16^ and with the *in*-*vitro* results (see above), suggesting the on-target effect. Talazoparib appears to also have reduced PARP1 levels and increased γH2AX. The combination further reduced FASN and PARP1 expression and further increased γH2AX level.

To determine the treatment effect on *in*-*vivo* BRCA1 expression, we performed western blotting analysis. As shown in Figure 6F, BRCA1 expression was reduced by 5HLS and talazoparib as a single agent and further reduced by the combination. We also analyzed the level of cleaved PARP1 as an indicator of apoptosis. Figure 6F showed that both 5HLS and talazoparib alone induced production of cleaved PARP1, indicating induction of apoptosis. The combination further increased the PARP1 cleavage, supporting the synergistic inhibition effect. γH2AX was also analyzed using western blotting, showing its induction by single agents and the combination (Fig. 6F), consistent with the immunohistochemistry data shown above. These findings support the *in*-*vivo* combination synergism between 5HLS and talazoparib.

## Discussion

Our previous findings on FASN regulation of PARP1 expression and PPI inhibition of FASN activity prompted us to test the hypothesis that PPIs may synergize with PARPi, creating an artificial synthetic lethality in BRCA1 wild-type TNBC cells and, thus, potentially expanding PARPi utility. Indeed, we showed in this study that inhibiting FASN using 5HLS or lansoprazole synergized with PARPi in both BRCA1 mutant and wild-type TNBC cells. This synergism is consistent with the fact that FASN contributes to PARPi resistance in these TNBC cells. These findings suggest that the combination of PARPi and PPIs or FASN inhibitors may create an artificial synthetic lethality for treating TNBC without consideration of BRCA germline mutation and, thereby, expanding PARPi utility to a broader population of TNBC patients.

**Figure 7:**
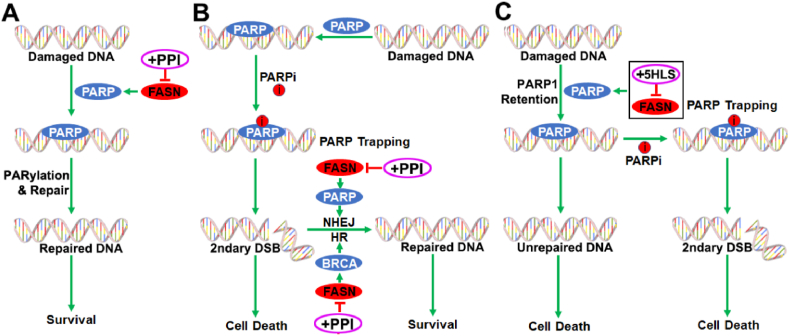
Model of PPI and PARPi combination synergy. **(A)** FASN regulation of PARP1 expression and its inhibition by PPI in DNA damage repair and cancer cell survival. **(B)** FASN in repair secondary double-strand DNA break (DSB) by regulating PARP1 and BRCA1 and its inhibition by PPI therein. **(C)** PARP1 retention in chromatin due to inhibition of FASN by 5HLS, facilitating PARP1 trapping by PAPRi.

FASN has been shown to up-regulate PARP1 expression, contributing to increased NHEJ repair of double-strand DNA breaks and, thus, resistance to DNA damage-induced cell death^32^ and inhibiting FASN expression or activity sensitizes cancer cells to these treatments^12^^,^^16^ (Fig. 7A). The finding that FASN increases PARPi resistance and inhibiting FASN function using 5HLS or lansoprazole synergizes with PARPi may stem from their effects on NHEJ repair of secondary DNA damage induced by PARPi trapping (Fig. 7B).

Our finding that 5HLS enhances PARP1 binding to chromatin while reducing its overall expression is intriguing. This suggests that inhibiting FASN may help recruit or retain PARP1 on chromatin while its expression is down-regulated. While this effect would enhance PARP1 trapping by PAPRi, leading to synergy with PARPi (Fig. 7C), it creates a conundrum and is inconsistent with our observation that 5HLS inhibits DNA damage repair activity, as shown previously^16^ and in this study. It is also peculiar to find that FASN up-regulates PARP1, leading to PARPi resistance, yet simply up-regulating PARP1 expression causes PARPi sensitivity due to PARP1 trapping. These findings also suggest that PARPi resistance is more complex than previously anticipated and that FASN function in PARPi-resistant cells may be more complex than just regulating PARP1 expression and DNA damage repair.

While the above conundrum could not be resolved currently, it is possible that a reduction in total PARP1 by inhibiting FASN reduces DNA damage repair activity, leading to accumulation of damage that signals for more PARP1 recruitment in a feedback mechanism. It is also possible that PARP1 palmitoylation affects its catalytic activity and retention. Previously, it was found that PARP1 palmitoylation reduced its retention and increased PARPi resistance.^33^ Considering FASN's role in palmitate production and protein palmitoylation,^34^, ^35^, ^36^, ^37^, ^38^ it is tempting to speculate that FASN not only regulates PARP1 expression but also regulates its palmitoylation. It is possible that the palmitoylated PARP1 is catalytically active and performs normal PARylation and DNA-binding dynamics for damage repair (Fig. 7A, B). The un-palmitoylated PARP1 may have prolonged retention without repairing DNA damage, causing cell death and sensitivity to PARPi (Fig. 7C). These speculations need to be tested in future studies, and we are working toward this effort.

FASN may also regulate the expression of other genes that contribute to PARPi resistance. One example is BRCA1 in the BRCA1 wild-type TNBC cells (Fig. 7B). By up-regulating BRCA1 expression, FASN effectively helps BRCA1 wild-type TNBC cells to bypass PARPi trapping-induced cytotoxicity, resulting in resistance to PARPi in these cells. Thus, inhibiting FASN function further contributes to sensitization to PARPi and synergism between FASN inhibitors and PARPi. However, it is currently unknown how FASN regulates BRCA1 expression. The finding that excess exogenous palmitate was able to rescue BRCA1 expression from 5HLS inhibition suggests that the mechanism of FASN regulation of BRCA1 expression is likely related to FASN function in palmitate production. Further studies are necessary to determine if BRCA1 up-regulation by FASN is due to BRCA1 palmitoylation and stabilization or due to increased gene transcription. In a recent study, it was found that FASN regulated p65 expression by destabilizing p65 via a palmitate-dependent phosphorylation-mediated isomerization mechanism.^19^

It is, however, noteworthy that PARPi sensitivity did not correlate with the BRCA1 proficiency in TNBC cell lines. This observation is consistent with previous findings.^26^^,^^39^ Indeed, many factors contribute to PARPi resistance or sensitivity,^40^ including loss of 53BP1, RIF1, or shieldin complex components (SHLD1/2/3), and Sky or SIK2 expression that allow DNA end resection restores or contributes to homologous recombination repair,^41^, ^42^, ^43^, ^44^, ^45^ restoration of replication fork protection due to loss of PTIP, MUS81, EZH2, or RADX,^43^^,^^46^^,^^47^ in BRCA mutant cells, as well as overexpression of ABC transporters such as ABCC1.^48^, ^49^, ^50^ It is unclear if FASN-induced PARPi resistance relates to any of these known PARPi resistance factors. However, we previously have shown that FASN does not regulate the expression or activity of ABC transporters, including ABCG2,^10^ suggesting that ABC transporters are unlikely to mediate FASN-induced PARPi resistance. Since NAD is required for PARP1 to function, while NADPH is required for FASN and the homeostatic regulation of NAD(H) and NADP(H) is essential for cellular metabolism,^51^ it is tempting to speculate that FASN may also regulate PARPi response by impacting the NAD-NADPH equilibrium.

Identification of lansoprazole as a representative PPI and its metabolite, 5HLS, as synergizing agents for PARPi to overcome PARPi resistance and to expand PARPi utility on TNBC, suggests a potential new clinical application of two FDA-approved drugs (PPIs and PARPi) in combination treatments of TNBC. Recently, it was shown in a randomized multi-center phase 2 trial that high-dose omeprazole nearly doubled the pathological complete response of TNBC patients to the standard-of-care chemotherapy with adriamycin, cyclophosphamide, and taxanes, and reduced about 50 % production of free fatty acids in the tumors of these patients.^52^ It is noteworthy that PPIs are short-lived, and the effect in the trial is remarkable. Our follow-up studies indicated that the PPI metabolites, including 5HLS with similar structures to their parent compounds, may continue to act on FASN to exert the long-lasting effect on TNBC cells.^16^ Interestingly, the sulfide derivatives of PPIs, such as 5HLS, do not inhibit proton pumps,^53^ suggesting that the synergism of PPIs, including lansoprazole and 5HLS, with PARPi is likely due to its inhibition of FASN, not proton pumps. This conclusion is supported by our genetic manipulation of FASN expression and the rescue experiment using palmitate.

In summary, we demonstrated in this study that lansoprazole, as a representative of PPI and its metabolite, 5HLS, synergized with PARPi by inhibiting FASN activity, leading to synergistic inhibition of NHEJ repair activity, accumulation of DNA damage, PARP1 trapping, and apoptosis. We also showed that 5HLS inhibited BRCA1 expression by inhibiting FASN in a palmitate-dependent mechanism, contributing to the synergy in BRCA1 wild-type TNBC cells. Further studies in targeting FASN using PPIs and their metabolites will likely lead to a novel approach to expand the utility of PARPi in treating TNBC without BRCA germline mutation.

## CRediT authorship contribution statement

**Sophia Josephraj:** Writing – original draft, Validation, Methodology, Formal analysis, Data curation. **Chao J. Wang:** Formal analysis, Data curation. **Qingbin Cui:** Resources. **Zizheng Dong:** Supervision, Methodology, Investigation. **Jing-Yuan Liu:** Writing – review & editing, Writing – original draft, Resources, Methodology, Funding acquisition, Formal analysis, Data curation. **Jian-Ting Zhang:** Writing – review & editing, Writing – original draft, Supervision, Resources, Project administration, Methodology, Investigation, Funding acquisition, Formal analysis, Conceptualization.

## Funding

This work was supported in part by the National Institutes of Health (NIH) grant 1R01 CA288278 (to J.Y.L. and J.T.Z.).

## Conflict of interests

The authors declared no conflict of interests.
