# Supplementary material for: Targeting fatty acid synthase to overcome PARP inhibitor resistance and to create an artificial synthetic lethality for triple-negative breast cancer
Source: Genes Dis. 2025 Aug 20;13(1):101817. doi: 10.1016/j.gendis.2025.101817 (PMC12557586; doi:10.1016/j.gendis.2025.101817)
Supplement: Multimedia component 1 [file mmc1.pdf]

Supplemental Materials

Targeting fatty acid synthase to overcome PARP inhibitor resistance and to create an artificial synthetic lethality for triple negative breast cancer

Sophia Josephraj[1], Chao J. Wang[1] Qingbin Cui[1], Zizheng Dong[1], Jing-Yuan Liu[1,2] and Jian-Ting Zhang[1*]

[1]Department of Cell and Cancer Biology, University of Toledo College of Medicine and Life Sciences, Toledo, OH 43614.

[2]Department of Medicine, University of Toledo College of Medicine and Life Sciences, Toledo, OH 43614.

*To whom correspondence should be addressed at jianting.zhang@utoledo.edu.

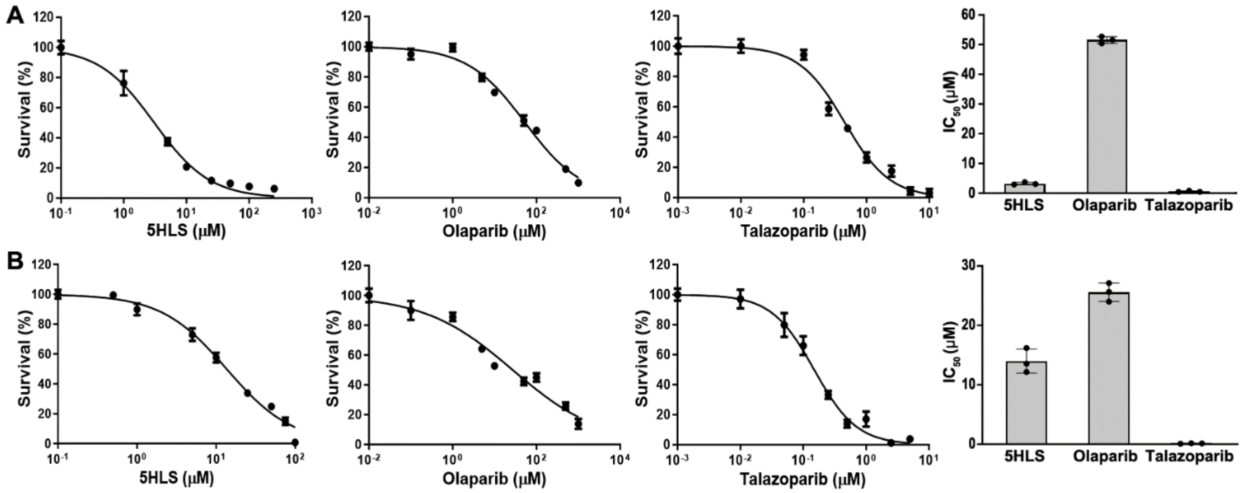

Figure S1. Dose-dependent survival of MDA-MB-231 (A) and MDA-MB-436 (B) cells as determined using methylene blue survival assay and the derived IC_{50} values of 5HLS, olaparib, and talazoparib from survival curves (n=3).

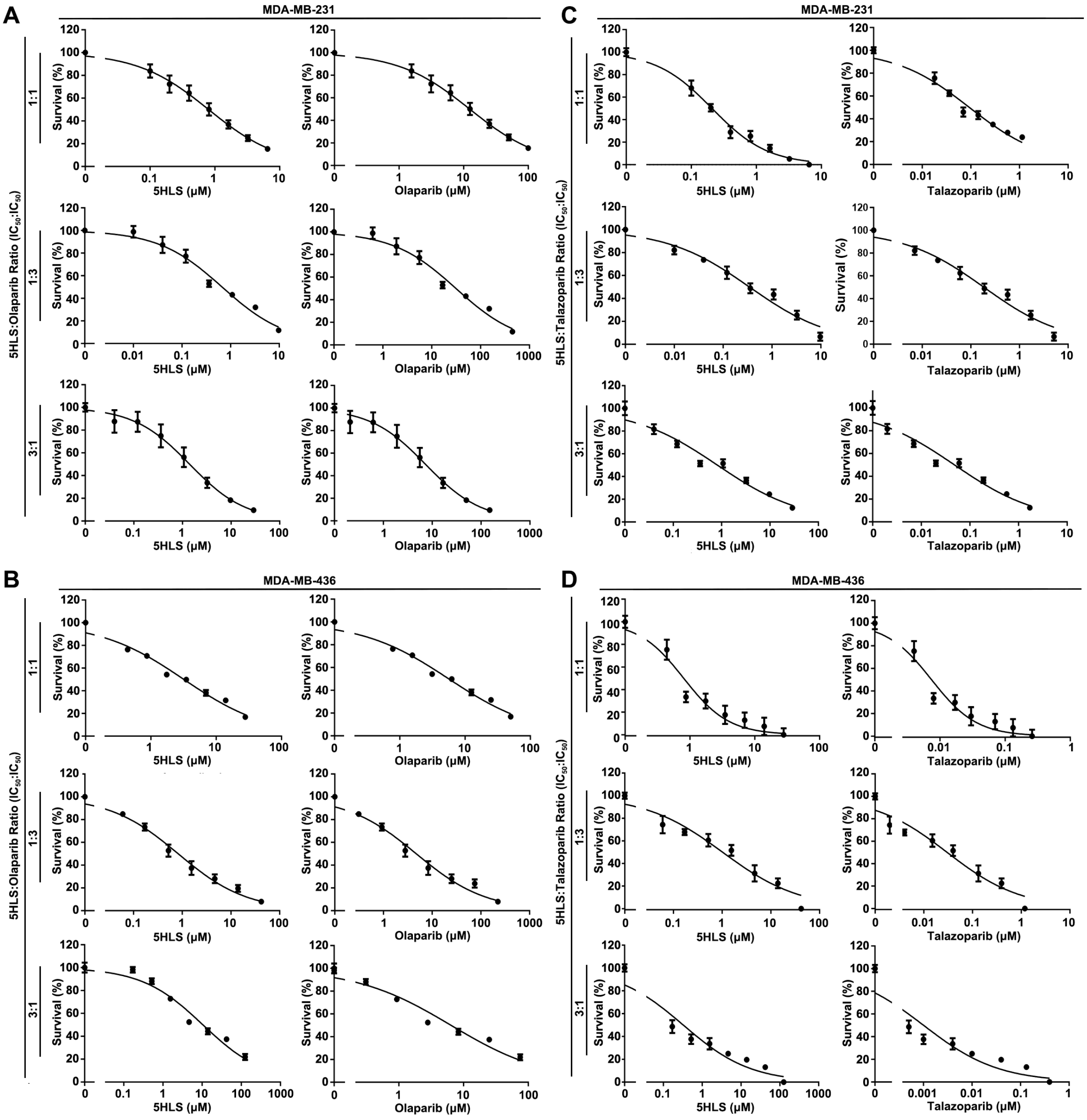

Figure S2. Combination analysis of 5HLS with olaparib (A and B) and talazoparib (C and D) was performed with three different ratios, followed by methylene blue survival assay to determine cytotoxicity in MDA-MB-231 (A and C) and MDA-MB-436 (B and D) cells.

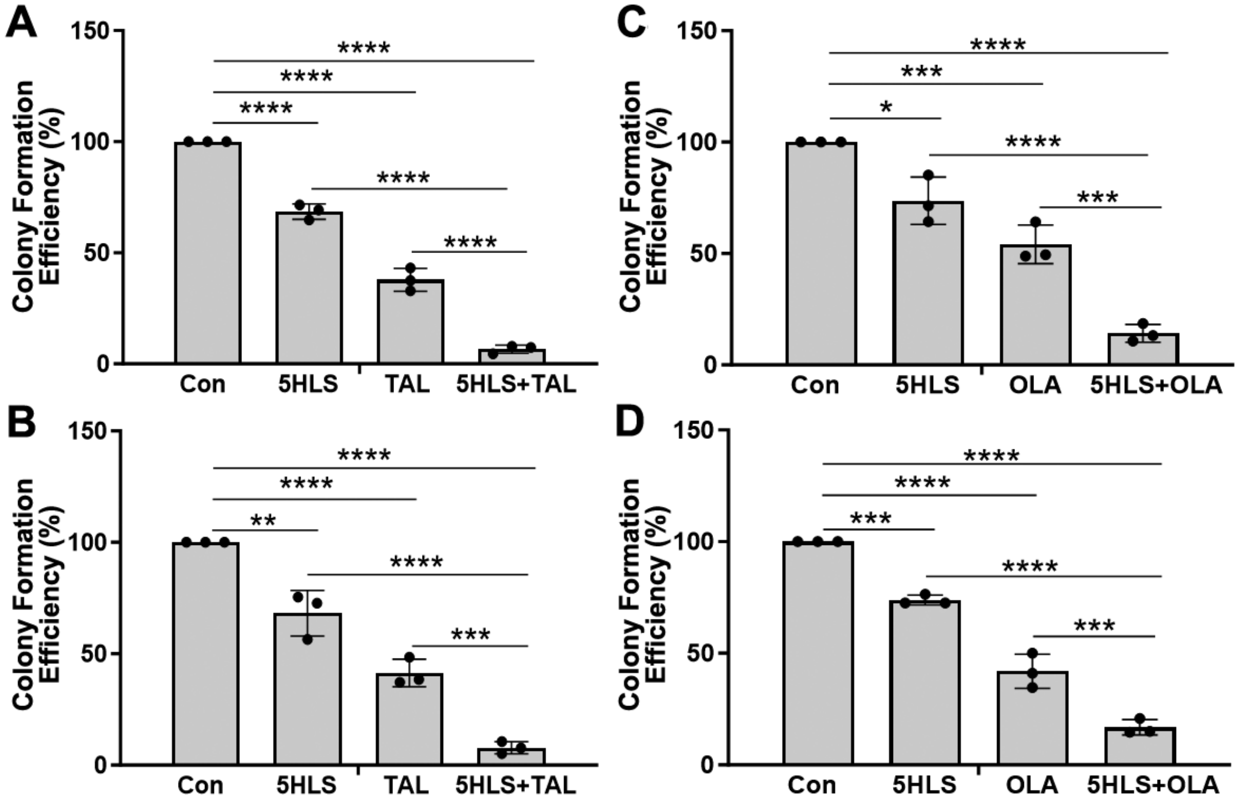

Figure S3. Colony formation assay in MDA-MB-231 (A, C) and MDA-MB-436 (B, D) cells treated for 7-10 days with the indicated drug treatments. Con, Control; Ola, Olaparib; Tal, Talazoparib; 5HLS, 5-hydroxy lansoprazole sulfide. n=3, ****$p < 0.0001$, ***$p < 0.001$ **$p < 0.01$, *$p < 0.05$.

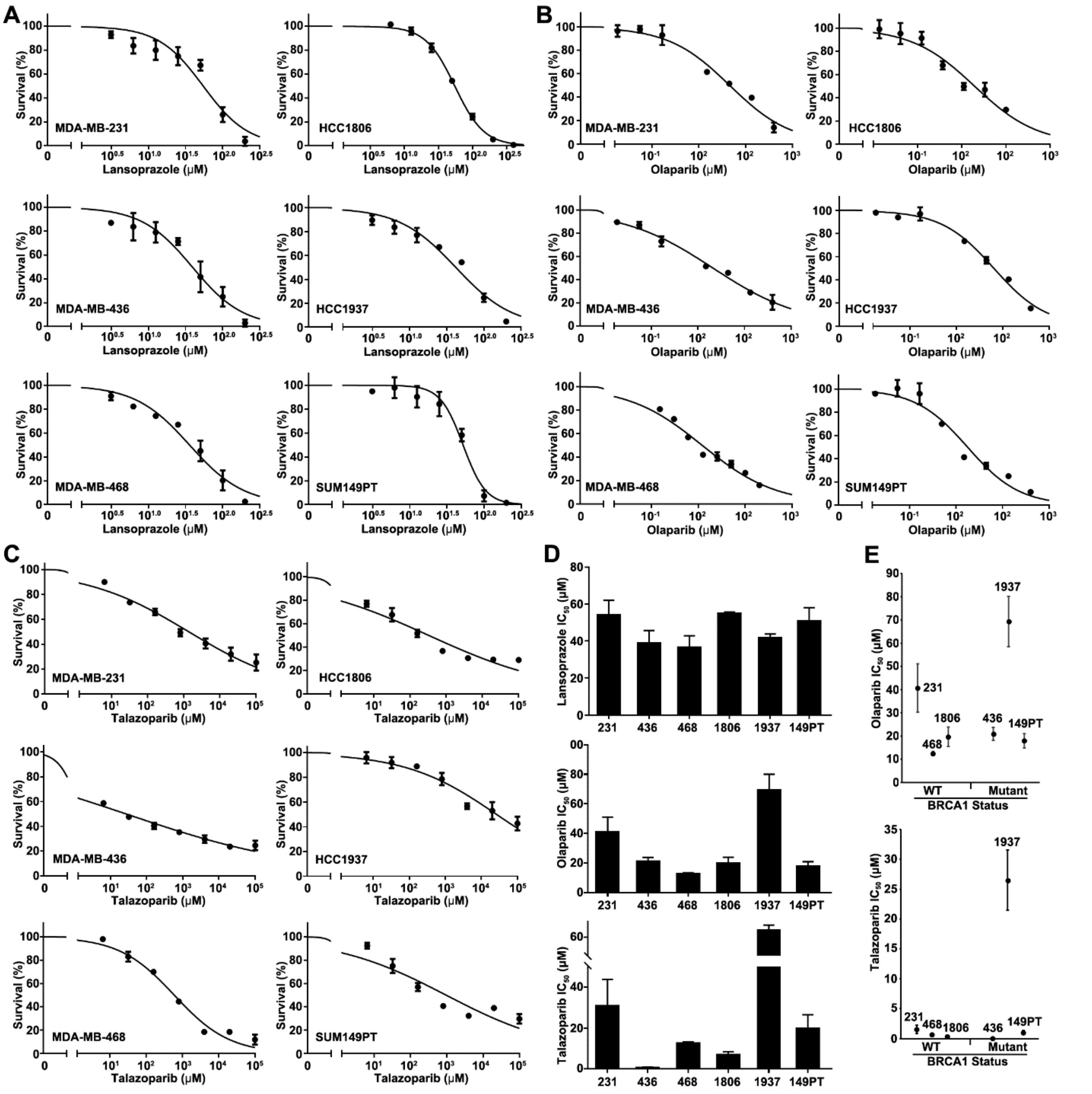

Figure S4. Dose-dependent survival assays using methylene blue staining were performed to determine the IC50 values of lansoprazole (A), olaparib (B), and talazoparib (C) in TNBC cell lines. Panels D and E summarize the IC50 values across TNBC cell lines. 231, MDA-MB-231; 436, MDA-MB-436; 468, MDA-MB-468; 1806, HCC1806; 1937, HCC1937; 149PT, SUM149PT. n-3.

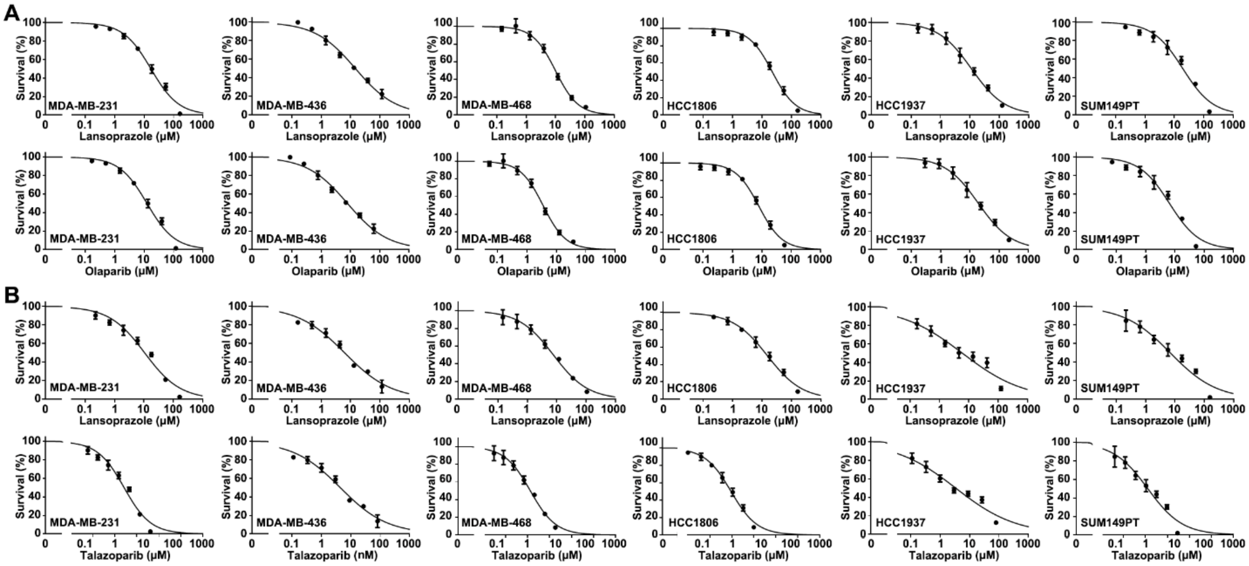

Figure S5. Combination effects of lansoprazole with olaparib (A) and talazoparib (B) in TNBC cell lines as determined using methylene blue survival assay.

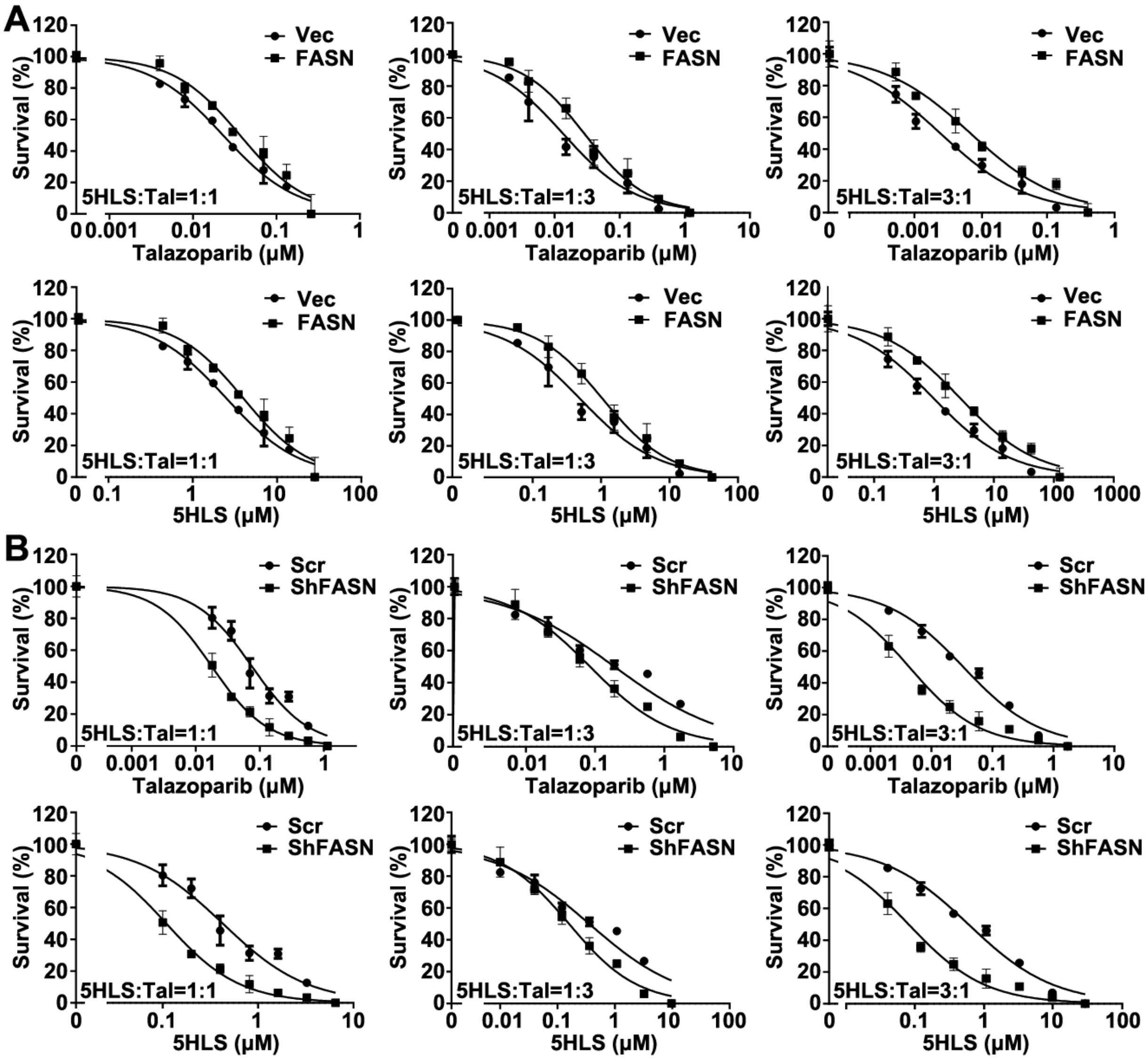

Figure S6. The synergistic effect of 5HLS and talazoparib on survival of MDA-MB-436 cells with stable overexpression of ectopic FASN (FASN) or vector-transfected control (Vec) cells (A) and MDA-MB-231 with FASN knockdown (ShFASN) or scrambled shRNA-transfected control (Scr) cells (B) as determined using methylene blue survival assay.

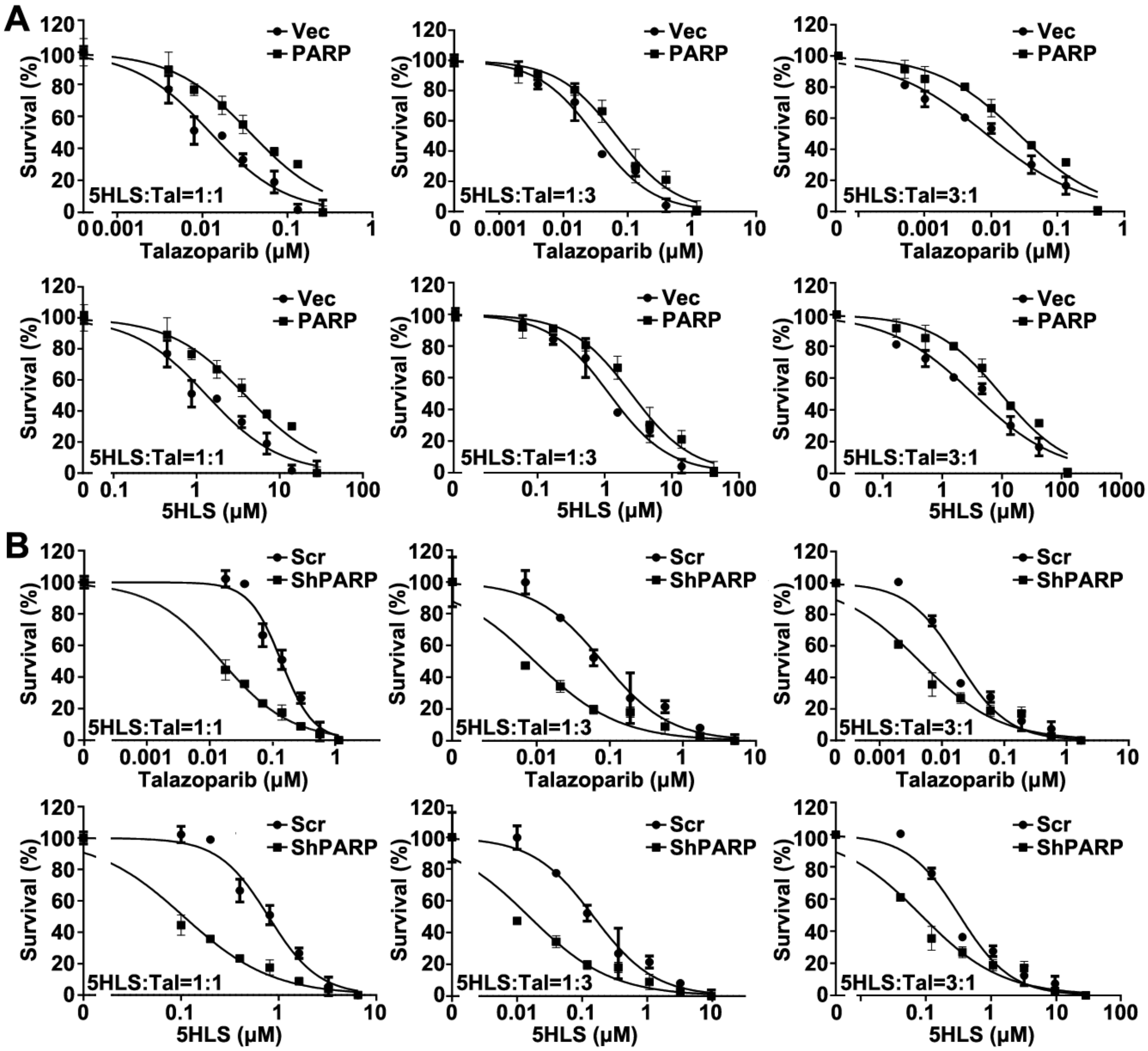

Figure S7. Combination effect of 5HLS and talazoparib on survival of MDA-MB-436 cells with stable overexpression of ectopic PARP1 (PARP) or vector-transfected (Vec) control cells (A) and MDA-MB-231 with stable PARP1 knockdown (ShPARP) or scrambled shRNA-transfected (Scr) control cells (B) as evaluated by the methylene blue survival assay.
